# T cell and bacterial microbiota interaction at intestinal and skin epithelial interfaces

**DOI:** 10.1093/discim/kyad024

**Published:** 2023-11-25

**Authors:** Damian Maseda, Silvio Manfredo-Vieira, Aimee S Payne

**Affiliations:** Department of Dermatology, Perelman School of Medicine, University of Pennsylvania, Philadelphia, PA, USA; Department of Dermatology, Perelman School of Medicine, University of Pennsylvania, Philadelphia, PA, USA; Department of Dermatology, Perelman School of Medicine, University of Pennsylvania, Philadelphia, PA, USA

**Keywords:** T cells, microbiota, epithelia

## Abstract

**Graphical Abstract:**
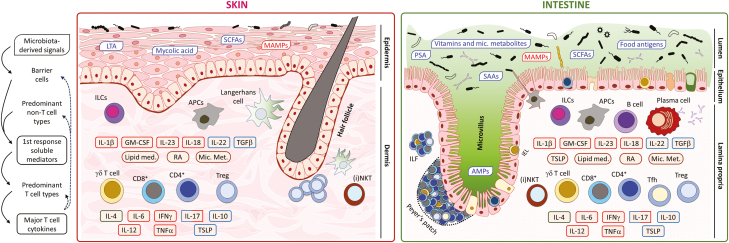

## Introduction

Our bodies are in continuous contact with diverse microorganisms, with most exposure occurring at epithelial sites. The skin and digestive tract epithelia are two of the biggest surfaces that provide this interaction, with even further enlarged contact areas due to epithelial invaginations like villi and microvilli in the intestines. Each barrier site contains an array of different cell types, including epithelial cells directly interacting with the environment, stromal cells supporting tissues, innervating neurons controlling motility and pain, and diverse immune cells. Barrier site T cells reside within or in close interaction with their neighboring epithelial surfaces, which house microbiota populations in their most external layers and continuously present microbiota-derived antigens to T cells [1–5]. In a healthy host, a continuous cross-talk among all barrier cells and the microbiota is necessary to achieve several concomitant goals: (i) protect against external pathogens, (ii) establish and maintain tolerance, and (iii) trigger repair programs to re-establish homeostasis when these barriers are compromised.

Others have reviewed the skin or intestinal microbiota composition and how they affect health and tissue-resident immune cells [6–9]. This review will focus on the impact of the skin and intestinal-localized bacterial microbiota on T-cell biology, highlighting both commonalities and differences across epithelia given their function, structure, and the microenvironmental conditions they provide for commensal microorganisms and their dwelling T cells. We will then discuss the types of T cells that inhabit the skin and intestinal epithelial barriers, covering how these environments can differentially activate resident T cells and enable them to be at the first line of defense while generating and maintaining tolerance to self and commensal bacterial microbiota.

## The skin and intestinal epithelia constitute unique niches for T cells

### Barrier tissue architecture enables local interaction of the microbiota and the immune system

**Figure 1:**
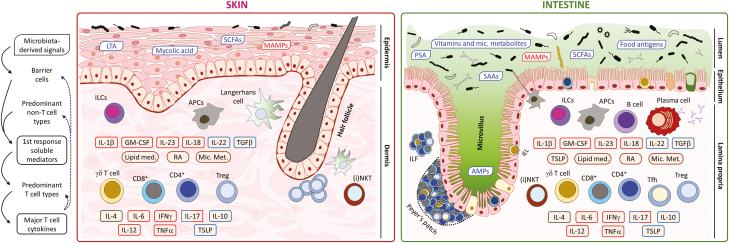
Soluble signals from the microbiota and T cells in the skin and intestinal barriers. Skin and intestinal bacteria present in the outermost layers of the barrier provide antigens and other signals. The skin epidermis and the intestinal epithelial monolayer are the first cellular structures to encounter our microbiota and can generate an array of signals in response. Many other cell types like mesenchymal cells, APCs, and ILCs reside in the dermis or lamina propria and can also release soluble mediators affecting T-cell function when sensing microbial triggers. T cells will integrate signals to modulate their function and cytokine profiles (continuous downward arrows). Cytokines produced by barrier-resident T cells can then have feedback effects on their host tissue (discontinuous upward arrows). Indicated are the main soluble signals involved in theses microbiota-T-cell circuits, with colors indicating a preference for these signals to be blue = protective/tolerant and red = inflammatory/pathogenic. APC: antigen-presenting cell, RA: retinoic acid, SCFA: short-chain fatty acids, MAMPs: microbial-associated molecular patterns, LTA: lipoteichoic acid, PSA: polysaccharide A, SAA: serum amyloid A proteins, AMP = anti-microbial proteins, ILF = isolated lymphoid follicle.

The intestinal barrier consists of a continuous epithelial monolayer with interspersed non-epithelial cells, sometimes with additional intra-epithelial immune cells in selected locations. Under this monolayer resides the lamina propria (LP), a connective tissue that houses different cell types, including many of the immune system. In contrast, the skin barrier comprises a stratified squamous epithelium from the external stratum corneum down to the basement membrane zone, which overlies the dermis and subcutaneous fat. The differing epithelial structures are associated with the development of different bacterial consortia and even unique structures like biofilms. These microbiotas integrate to different extents with the local barrier tissue, enabling the generation of specialized host tissue–microbiota systems. Immune and non-immune cells at both barrier sites scrutinize antigens that can be presented *in situ* or taken to their respective draining lymph nodes (LN). The intestinal milieu displays several types of organized tertiary lymphoid structures (TLS) like Peyer’s patches, isolated lymphoid follicles, and cryptopatches [10] (Fig. 1). The skin provides less abundant, but also specialized, immune-interaction hubs like the hair follicles (HF) within pilosebaceous units [11] or ectopic lymphoid structures. These niches reproduce some features of their intestinal equivalents, especially in inflamed skin [12]. Interestingly, the HF is an immune-privileged site, as it expresses Fas ligands but lacks Major Histocompatibility Complex (MHC) class II expression. Furthermore, HFs are protected from T-cell cytotoxicity while quiescent [13] and are surrounded by an extracellular matrix (ECM) containing immune-suppressive components [14]. Apart from presenting antigens, many of these structural barrier cells can also impact T-cell function employing soluble molecules like cytokines, microbiota-driven metabolites, and lipid mediators of inflammation (Fig. 1). T cells can seed and recirculate back and forth from barrier tissues, with the establishment of T-cell residence marking the beginning of a dynamic orchestration of T-cell responses to commensals.

### T-cell residence and activation at barrier sites

**Figure 2:**
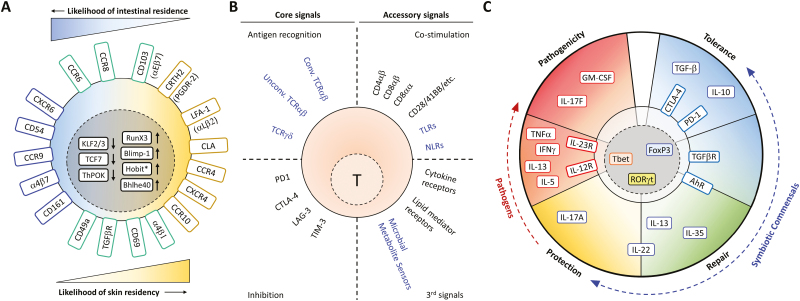
Activation, residence, and response of T cells at barrier sites. (A) Surface molecules and transcription factors invovled in recruitment and retention in either the intestinal or the skin milieu. (B) Recognition of microbiota-derived antigens and other signals that modulate T-cell activation derived from the barrier environment. Depicted are different T-cell-intrinsic and -extrinsic signals sourced by the microbiota, divided in core and accessory mediators of T-cell activation. Integration of all these signals leads to the final T-cell response. Highlighted in blue are receptors that can directly bind microbial components or metabolites. (C) Main transcription factors, surface receptors, and cytokines that influence the spectrum of responses from barrier T cells into tolerogenic, pathogenic, protective, or reparative programs. Placements are not necessarily exclusive, as marker and cytokine patterns can be shared depending on the kind of T cell and the type and time of response. *Expressed only in murine cells.

T-cell homing and/or residence in the intestine or skin is associated with the expression of chemokine receptors like CCR9 for the gut and CCR4/8 for the skin. While integrin α4β7 and CCR9 are a hallmark of intestinal habitation, skin homing is preferentially bestowed by CLA, α4β1 (LFA-1), and CCR4 [15, 16]. Importantly, the skin and intestinal barrier tissues share several of the ligands for these T-cell homing surface molecules, although to different degrees (Fig. 2A). The probability of T-cell residence is not unequivocally determined by these surface markers but is manifested as a spectrum pattern, and likely depends on additional signals yet to be discovered.

T-cell activation at barrier sites is largely driven by antigens expressed locally or in neighboring lymphoid organs. Barrier sites contain TLS, which promotes adaptive immunity, but are canonically considered less specialized for antigen-driven priming of T cells than secondary lymphoid organs such as the spleen and draining LNs. Microbial antigens are presented to T-cell receptors (TCRs), while antigen-agnostic microbe-related signals like microbial-associated molecular patterns (MAMPs) or bacterial metabolites are sensed through toll-like receptors (TLR), NOD-like receptors (NLR), and other microbial metabolite receptors (Fig. 2B). Barrier antigens are offered by antigen-presenting cells (APCs) like epithelial cells, B cells, or monocytic-derived cells, which can become highly specialized barrier antigen-presenting dendritic cells (DC) [4, 16–18]. Other atypical APCs like goblet cells, M cells, or neutrophils are present in the intestines [17], while the skin contains distinct site-specific APCs like Langerhans cells (Fig. 1). Human skin Langerhans cells can express non-canonical antigen-presenting molecules like CD1a that can control inflammatory responses through induction of IL-17 and IL-22 in local T cells [19], and CD1a-autoreactive T cells can mediate cutaneous allergic and inflammatory responses [20]. The immune cells that co-habit barrier sites and have a high degree of interaction with T cells must trigger and maintain responses to microbial threats while simultaneously collaborating in setting up a tolerogenic state to the local commensal microbiota.

A variety of microbiota metabolites affect T cells, including short-chain fatty acids (SCFA), vitamin B2 metabolites, deoxycholic and lithocholic acids, secondary bile acids, and polyamines [21] (Fig. 1). As one example, tryptophan derivatives can be sensed through the aryl hydrocarbon receptor (AhR). AhR is widely expressed in the intestines and has a prominent role during regeneration following tissue injury [22]. Tryptophan can be metabolized to indole-3-lactic acid, a ligand for AhR, by the murine intestinal commensal *L. reuteri*, and this AhR signal is responsible for downregulation of the transcription factor ThPOK in T cells and consequent induction of CD4+CD8αα+ intraepithelial lymphocytes (IELs), although the mechanism for this induction is currently unclear [23]. AhR also mediates type 3 innate lymphoid cell (ILC3) and Th17 induction in the murine intestine, and the absence of AhR signaling allows for increased susceptibility to enteric infection [24]. The catabolism of tryptophan by intestinal microbiota to indole-3-ethanol, indole-3-pyruvate, and indole-3-aldehyde can prevent increased gut permeability in a mouse model of colitis [25]. Likewise, AhR signaling of the skin microbiota in keratinocytes protects them from barrier damage and infection [26]. The effects of barrier microbiota on host T cells can be due to the properties of single microbes or community-level properties of the whole barrier microbiota. This becomes especially relevant when considering metabolites generated only by specific microbiota species consortia that partially share metabolic pathways. Further discussion of how the microbiota can influence resident memory T-cell homing appears later in this review in the section entitled "Tissue resident memory T cells (TRMs): the local guys are ready for a fight". Collectively, the emerging data indicate that the gut and skin epithelia provide specialized microenvironments for bacterial microbiota and immune cell interaction and that niche-specific commensal microbiota influence T-cell function at these sites.

## Types of T cells in the skin and intestinal barriers: a mixture of conventional and unconventional flavors

### Diverse types of T cells are necessary at barrier sites to establish homeostasis and protection against pathogens in the presence of microbiota

As immune-rich organs, barrier sites host T cells belonging to common ontogenically defined types like conventional TCRαβ+ single-positive CD4+ or CD8+ cells and TCRγδ+ cells. The skin and intestinal tissues are also especially rich in unconventional T cells that often exert innate-like and rapid-response properties. Such cells include TCRγδ+, NKT, and iNKT cells, which bear invariant or semi-invariant TCR chains [27]. The intestines also house unique populations like mucosal-associated T cells (MAIT), TCRαβ+CD4−CD8−T cells, TCRαβ+CD8αα+, and CD4+CD8αα+ double-positive T cells (the latter being absent in gnotobiotic mice). Some are associated with specific TLS, but most intestinal resident T-cell pools diffusely populate the intestines and skin in the LP or dermis. In the intestine (especially in the small intestine), T cells are also present within the epithelial layer as IELs.

In mice, the skin generally contains more CD8+ and TCRγδ+ T cells than the intestine. Conversely, the gut houses proportionally more CD4+ and unconventional T cells. This is likely due to the impact of their microenvironment, including the type of co-habiting APCs and the interactivity with the local microbiota. Much evidence has accumulated about the role of the microbiota in T-cell function, with many investigations linked to CD4+ IL17+ (Th17) and regulatory (Treg) T cells [21, 28], discussed in further detail below. The relevance of the microbiota in CD8+ T-cell function is less known, but recent studies show that a consortium of 11 bacterial strains from healthy human donor feces can induce IFN-γ-producing CD8+ T cells in the intestine and confer protection against *Listeria monocytogenes* infection [29]. Barrier epithelial cells or APCs are a predominant source of cytokines like IL1β, IL-18, IL-22, IL-23, GM-CSF, and TGFβ, which strongly influence T-cell responses (Fig. 2c). The intestinal and skin epithelia must balance homeostatic regulation to maintain structural harmony with highly proliferative and regenerative capacities when responding to injuries and insults that compromise the barrier. In this context, type-2 T-cell responses in the skin and gut are linked not only to defense against parasites but also to regenerative responses [30]. Barrier IL-33, IL-25, and TSLP, which can drive a dominant part of type-2 responses [31], are also part of epithelial reparative programs. Additionally, T-cell surveillance and defense at barrier sites rely on cytotoxic and/or pro-inflammatory programs to battle pathogens, but when dysregulated, this can be a cause of tissue damage, as happens in skin and intestinal autoimmune and inflammatory conditions. Microbiota-reactive T cells will hence influence their local barrier environments, including bystander T cells (largely driven by Th2, Th17, and Treg cells) that collectively can mount a memory response to a pathogen insult, trigger targeted or off-target cytotoxic responses, or contribute to tissue homeostasis or repair. Dissecting the mechanisms by which the microbiota elicits optimal T-cell responses to pathogens while maintaining tissue integrity and tolerance remains an active area for current and future investigation.

Unconventional T cells can sense microbes indirectly by recognizing microbial metabolites [21]. Microbial metabolites control different aspects of thymic development of T cells in barrier sites: In mice, thymic development of MAIT cells is governed by bacterial products captured by non-classical Ag-presenting major histocompatibility complex class I-related (MR1) molecules [32], and in the case of the skin, such induction is restricted to a specific early-life window in response to cutaneous riboflavin-synthesizing commensals [33]. The microbiota does, however, retain the capacity to modulate barrier T cells in the adult. For example, the intestinal commensal *Lactobacillus reuteri* can induce CD4+ CD8αα+ IELs, which differentiate from IEL CD4 + T cells sensing tryptophan derivatives through the aryl hydrocarbon receptor (AhR) [23]. Interestingly, while AhR expression is paramount for unconventional T cells, it is also critical for Th17 and Treg cells [22]. Additionally, β-hexosaminidase, a conserved enzyme across commensals of the *Bacteroidetes* phylum, was recently identified as a driver of CD4+ IEL T-cell differentiation. Importantly, such β-hexosaminidase-reactive T cells were able to confer protection against intestinal inflammation in a mouse model of colitis [34]. TCRγδ+ T cells constitute a different lineage that acts as first responders due to their restricted reactivities and quickness to engage and respond [35]. In the intestine, TCRγδ+ T cells preferentially reside in the small intestine, and TCRγδ+ T cells that produce IL-17 are generally protective against pathogens [36]. Specific intestinal commensals like *Bacteroides* species are required for maintaining mouse TCRγδ+ IL-1R1+ cells, which are a potential source of IL-17 that can be activated by IL-23 and IL-1 [37]. In murine skin, basal keratinocytes expressing the oxysterol catabolic enzyme cholesterol 25-hydroxylase maintain TCRγδ+IL17+ cells, and oxysterols in the diet can increase psoriatic inflammation driven by these cells [38]. Additionally, skin-resident γδ T cells can activate an IL-17A/HIF-1A-dependent repair response in epithelial cells [39]. Notably, TCRγδ cells are present in significant proportions in homeostatic conditions in murine skin but are less common in human skin, where they are also less likely to produce IL-17.

### Th17 cells: barrier guardians with high plasticity and autoimmune potential

T cells capable of IL-17 production (Th17) constitute a critical part of the immune system in barrier tissues [40–42]. IL-17 has five receptor members and six isoforms (A-F), with IL-17A being predominantly expressed and most studied. IL-17F (closest in sequence homology to IL-17A), but not IL-17A, can reduce the presence of Treg-inducing *Clostridium* cluster XIVa in colonic microbiota, and IL-17F drives intestinal pathology in a T-cell transfer mouse model of colitis [43]. In humans, serum IL-17A may reflect the active phase in ulcerative colitis, but not in Crohn’s disease [44], but whether there is such a division of responses in IL-17A and 17F in the skin is still not established. IL-25 (IL-17E) can also be induced by microbiota commensals in intestinal epithelial cells. IL-25 can inhibit the expression of macrophage-derived IL-23 and hence limit the expansion of murine Th17 cells in the intestine [45]. Once Th17 responses have been triggered, specific feedback signals can be delivered to the microbiota: For example, IL-22 induces the production of anti-microbial peptides [46], and specific anti-microbial peptides secreted by neutrophils like cathelicidin can also cause the differentiation of Th17 cells in the presence of TGFβ1 [47], which illustrates the complexity of the circuitry involved in Th17 cell responses to bacteria.

Many studies have demonstrated the critical relevance of IL-17 cytokines in protecting against microbial insults in the intestines and skin [37, 48]. However, how the microbiota regulates the relative contribution to infection, pathogenesis, or the inflammatory resolution of IL-17-producing T cells remains to be deciphered. This might be, to a great extent, due to the plastic nature of Th17 cells, which can switch or add cytokines to their repository depending on environmental triggers [49]. IL-6, IL-1β, TGFβ, and IL-23 are required for Th17 induction and maintenance to different extents [48, 50]. However, in inflammatory environments, while IL-23 is not sufficient for Th17 induction on its own [8], it is a critical regulator of Th17 pathogenicity, which is largely mediated by induction of IFNγ and/or GM-CSF [51, 52]. In addition, serum amyloid protein A, produced during acute inflammatory responses in the intestine, can also strongly imprint a pathogenic Th17 program that bypasses TGFβ requirements [53]. On the contrary, the production of IL-17 and IL-22 is critical for protection, especially against fungi or bacteria like *C. rodentium* [41]. In mice, epithelium-MHCII can limit the accumulation of commensal-specific Th17 cells and generate protection against commensal-driven inflammation [54]. However, ILC3 cells also express MHCII and regulate T-cell responses independent of IL-17A, IL-22, or IL-23 [55]. MHCII expression in ILC3s can directly induce cell death of activated commensal bacteria-specific CD4+ T cells, and microbiota-induced IL-23 can reversibly silence MHCII in ILC3s [3]. These examples also illustrate how cytokines directly induced in barrier cells by microbiota contribute to the local milieu, increasing the complexity of signal networks regulating specific T-cell outcomes. In the colon, T-cell reactivity to intestinal microbiota might be altered in patients with inflammatory bowel disease (IBD), as the presence of T and Th17 cells is increased in intestinal tissue isolated from IBD patients (but not healthy controls or IBD patients’ PBMCs) [56]. The plasticity of Th17 cells extends even to their capacity to suppress IL-17 while inducing IL-10 [57], or their colonization of Peyer’s patches to become Tfh cells, boosting IgA production [58].

The type of bacterial antigen and corresponding environmental cues are vital in guiding the specific Th17-cell response. Microbiota species-specific responses consistently promote IL-17 production in murine lamina propria CD4+ T cells [59]. Moreover, the TCR repertoire of Th17, but not other intestinal T cells, is shaped by segmented filamentous bacteria (SFB), illustrating how antigen specificity and T-cell effector functions can be matched [42]. Interestingly, the presence of SFB in mouse microbiota can induce intestinal bacteria to produce retinoic acid (RA, another non-antigen signal critical for T-cell biology at barrier sites), conferring a protective function against *Citrobacter rodentium* infection [60]. Conversely, some *Clostridia* species can impair RA levels by suppressing the expression of retinol dehydrogenase 7 (Rdh7) directly in IECs, which can, in turn, reduce IL-22 anti-microbial responses and enhances resistance to colonization by *S. typhimurium* [61]. In mice, SFB and *Escherichia coli* (EHEC) adhesion to intestinal epithelial cells are responsible for specific Th17 responses [40]. Interestingly, the effect of SFB on Th17 cells is linked to several autoimmune manifestations. For example, compared to germ-free mice, mice that harbor SFB are more susceptible to experimental autoimmune encephalomyelitis (EAE) [62], and SFB colonization can also drive arthritis in a murine model [63]. By contrast, the presence of SFB is strongly correlated with a diabetes-free state in non-obese diabetic mice [64]. Such contrast of pathogenic vs. protective responses highlights how sensitive to context are microbiota-T-cell interactions. Such disparities can be due to mouse models that very differently recapitulate disease pathogenesis in different locations but also because of the plasticity of Th17 responses. Several other commensals are also capable of inducing Th17 cells: The human symbiont bacterial species *Bifidobacterium adolescentis* can alone induce Th17 cells in the murine intestine and exacerbate pathological features of autoimmune arthritis in the murine K/BxN model [65]. In this context, mucosal-adapted *E. gallinarum* translocates to secondary lymphoid organs and the liver and may promote maladaptive Th17 responses in tissues and blood [66], contributing to extraintestinal autoimmunity. Another illustration of how commensals can promote or prevent disease in a context-dependent manner is the case of *H. hepaticus*, which can trigger colitis in mice deficient in IL-10 [67] but can also induce RORγt+FOXP3+ regulatory T cells that selectively restrain pro-inflammatory Th17 cells [68].

As noted for the intestine, the skin microbiota can also have divergent outcomes for skin Th17 cell responses, even with signals from the same bacterium. For example, *S. aureus* toxin A induces Th17 responses [69]. Additionally, skin inflammation caused by *S. aureus* induces the release of alarmins by keratinocytes that induce IL-17 production by T cells [70]. In contrast, LTA from *S. aureus* can induce T-cell anergy [71]. Protective Th17 responses are also paralleled in the skin, like during infection with *C. acnes*, generating a protective pathogen-specific cytotoxic Th17 program that includes the formation of neutrophil extracellular traps (NETs) [72]. Another Th17-protective skin response is guided by *S. epidermidis*, one of the major facultative anaerobe components of the bacterial skin ecosystem, which induces CD8+IL-17A+ T cells that home to the epidermis. Once in the skin, these CD8+IL17A+ cells enhance innate barrier immunity and limit pathogen invasion, with skin DCs mediating this effect in the absence of inflammation [18]. One apparent difference between intestinal and skin Th17 responses is that induction of Th17 in the skin depends on IL1R but is independent of IL23R [73]. However, local IL-23 is required for the proliferation and retention of skin-resident memory Th17 cells [74]. Interestingly, non-classical MHCI-restricted commensal-specific immune responses in the skin can drive antimicrobial T-cell responses together with tissue repair [75]. Supporting this, Harrison *et al*. found that skin commensal T-cell reactivity drives a predominant IL-17 response during homeostasis. However, in the context of tissue injury, alarmins like IL-1, IL-28, and IL-33 trigger a type-2 program that is superimposed, leading to concomitant tissue repair that is T cell IL-13-dependent [76].

Many other lines of evidence illustrate the relevance of IL-17 in human barrier health: Mutations in genes involved in the IL-17/IL-23 axis have been identified as risk factors for psoriasis [77] ankylosing spondylitis [78], and IBD [79]. Both IL-17 and IL-23 are targets for drugs used to treat inflammatory conditions, including skin and intestinal diseases [80]. Interestingly, targeting IL-17A itself was initially developed for IBD but resulted in exacerbated symptoms, and the approach has since then proven safe and effective for treating psoriasis and psoriatic arthritis [80, 81]. This emphasizes the delicate balance and role of cytokines depending on context and indicates that each cytokine’s protective versus pathogenic roles must be carefully considered for each tissue and disease. While it is unlikely that the differences in response in skin and intestinal tissues to current anti-IL17 family therapies are solely due to their local microbiota composition, the microbiota can strongly influence the IL-17/IL-23 axis and hence the outcome of T-cell responses during pathogenesis in barrier diseases. More research is therefore needed to reconcile and advance our knowledge to enable a safer and more effective transfer of immune-directed therapies into clinical practice, including a better understanding of how the local cytokine networks mediate crosstalk between microbiota and barrier tissues.

### Local Tregs: more than just suppression of undesired T-cell responses

Barrier site environments represent a challenge to Treg cells, as Tregs must establish and maintain tolerance to commensals while facilitating standard tissue-specific T-cell effector functions. In the intestine, Tregs are fundamental in controlling pro-inflammatory responses to microbial communities and diet [28, 82–88], as well as modulating epithelial tissue repair programs [89]. Tregs can derive from thymus precursors (tTregs) or be generated in the periphery (pTregs). Tolerance to gut microbes starts to be established during early life, but intestinal commensals are also critical to drive pTreg formation in the adult [1, 90, 91]. Tolerance to skin commensal bacteria is preferentially established in neonatal life with unique waves of activated regulatory T cells entering the skin [85]. Hence, most skin Treg cells belong to the tTreg-cell subset, while the intestine is richer in pTregs. Intestinal pTregs can be generated and function in the LP, the mLN, and TLS [90, 92]. Paradoxically, the microbiota is dispensable for the early stages of peripheral regulatory T-cell induction within murine mesenteric LN [93]. In contrast, skin Tregs accumulate preferentially in the vicinity of hair follicles [94]. Due to their respective environmental peculiarities discussed above, gut Tregs seem to have a bias for maintaining tolerance, while the role of skin Tregs is geared toward tissue regeneration.

Tregs can be assigned to control canonical types of immune responses when they co-express FoxP3 with either Tbet, GATA3, or RORγt, exerting suppressive capacity on their mirroring effector T-cell types [21]. In general, while GATA3+ Tregs develop in response to dietary antigens, RORγt+ Tregs are generated in response to the microbiota. *In vivo* studies reported that animals deficient in the FoxP3 intronic enhancer conserved nucleotide sequence 1 (CNS1) displayed microbiome dysbiosis and elevated type 2 immune response in the colon [95]. Mouse colonic GATA3 + Treg cells can express ST2, the receptor of the IEC-derived alarmin IL-33, where it promotes Treg function and adaptation to inflammatory environments. Strikingly, IL-23 can inhibit IL-33 responsiveness in T cells, counteracting its Treg-promoting role [89]. The lamina propria is also augmented for Treg cells with high expression of RORγt, with most of such cells displaying constitutive expression of cytotoxic T-lymphocyte-associated protein 4 (CTLA4) and inducible expression of IL-10, IL-35, and TGF-β [88, 90, 91, 96]. Several bacterial species can induce the generation of FoxP3 +RORγt+ T cells in gnotobiotic mice, and in murine models of intestinal autoimmunity driven by T-cell transfer, the absence of Th17-like Treg cells can exacerbate pathogenesis, indicating its protective function [96, 97]. The effects of the microbiota on Tregs can vary from the niche-population commensal level to single molecules expressed by specific phyla. For example, in mice, broad-spectrum antibiotics are more potent in the depletion of RORγt+ Treg cells than individual antibiotics, showing that community-level functions of microbes rather than individual microbial phyla have a larger effect on Treg cells [97]. Additionally, other barrier cells fundamental for antimicrobial defense, like ILC3 cells, can promote Treg RORγt+ cells in a microbiota-dependent manner [91]. Specific *Clostridia* lacking prominent toxins and virulence factors found in humans, like clusters IV, XIVa, and XVIII, can induce differentiation and expansion of Tregs in murine transfer experiments [98]. *Clostridium* XIVa populations are also increased in mouse models of colitis where there is a simultaneous expansion of colonic Tregs [43]. At a molecular level, polysaccharide A from *B. fragilis* mediates the conversion of CD4+ T cells into Foxp3+ Treg cells that produce IL-10 during commensal colonization, with TLR2-mediated signaling being necessary for both induction of Tregs and IL-10 expression. Furthermore, polysaccharide A can inhibit colitis development in mouse models [83]. Tregs are also capable of directly detecting other MAMPs, illustrated by how MYD88-deficient Tregs induce Th17 cell dysregulation and bacterial dysbiosis, which are linked to impaired Tfh generation in Peyer’s patches and intestinal IgA [99]. Such results also portray the continuous high degree of interconnectivity in the microbiota-immune cross-talk. RA also has a strong influence on Tregs [100], and CD161+ regulatory T cells responding to RA can support wound repair in intestinal mucosa [101]. In the skin, sensing of RA through RXRα receptors in keratinocytes regulates hair cycling, proliferation, and differentiation [102]. Tregs are also involved in skin epithelial stem cell differentiation [94], but the role of skin commensals in this context has not been addressed.

Akin to Th17 cells, Tregs also display some degree of plasticity. Treg malleability can induce loss of suppressive potential, although the relative relevance of this phenomenon is still under debate. Interestingly, upon migration to the intestinal epithelium, murine colonic lamina propria Tregs can lose Foxp3 expression and convert to CD4+ IELs in a microbiota-dependent manner [87]. The conversion of Treg cells into pathogenic Th17 cells has been described in mouse models of psoriasis, and Th17-like Treg cells have been found in the skin of psoriatic patients [103]. However, it remains to be determined whether Treg plasticity is a mechanism to restrict suppression in barrier milieus or if it can be causally linked to autoimmunity. TGFβ is required to generate Tregs and Th17 cells, and synergy between TGFβ and RA induces differentiation of CD4+Foxp3+ Treg cells [84]. Some microbiota members, like *C. butyricum*, can induce TGFβ1 in lamina propria dendritic cells [104]. In the skin, TGFβ controls migration of Langerhans cells [105], and activation of TGFβ by keratinocytes mediates antigen-specific circulating memory CD8+ T-cell responses [2]. However, how the microbiota impacts the skin to modulate T-cell responses through TGFβ is still largely unknown.

Sensing of metabolites produced by commensals can also have a significant impact on Tregs: recruitment and location of Helios+ Tregs in the gut is dependent on signals delivered through AhR on the intestinal epithelium [106], and microbiota-derived metabolites of bile acids can modulate colon RORγt+ Treg cells [86]. SCFAs, which are fermented from dietary fiber by the gut microbiota, strongly influence intestinal Treg cell responses [107–110]. The large intestine shows a considerable enrichment for SCFAs, promoting pTreg cell responses in the gut. In specific-pathogen-free mice, administration of SCFAs increases the number of colonic Treg cells. Although intestinal inflammation and pathology reduction were observed in mice and humans treated with SCFAs, it is unclear whether this is mediated preferentially by Treg cells [108]. Skin Treg cells might also be substantially affected by the local microbiota, which can ferment SCFAs from skin lipids and generate indoles with potent AhR activity [111].

Regulatory T cells can also mediate specific aspects of tissue reparative programs. In mice, IL-33 induces TGFβ1-mediated differentiation of Treg cells and promotes Treg-cell accumulation and maintenance in inflamed tissues, which they collaborate to repair [89]. Amphiregulin, a molecule involved in inflammatory repair responses [112], is found in murine Tregs, where it increases Treg suppressive function subset of Tregs through EGFR sensing [113]. In the skin, Tregs have been found to promote hair follicle regeneration by augmenting hair follicle stem cell proliferation and differentiation [94]. Murine skin Tregs also express high levels of GATA3, which skews them toward a T helper (Th) type 2 and enables them to suppress skin fibrosis [114].

### Tissue-resident memory T cells (TRMs): the local guys are ready for a fight

Most of our knowledge of T-cell biology originates from rodent studies and human T cells present in the blood, but a wave of recent research has reinvigorated the interest in T cells within tissues. Tissue-resident memory T cells (TRMs) are non-migratory T cells that persist in the absence of cognate antigen exposure, possess heightened effector functions, and protect against known pathogens in the tissues they inhabit [115, 116]. TRMs provide local surveillance and can generate local and systemic responses on pathogen rechallenge [117, 118]. Phenotypical markers of residence at the intestinal and skin barrier sites are well established, and while some of these molecules seem to be exclusive for a specific barrier site, others are shared to different degrees (Fig. 2A). While TRMs are defined by their location, TRMs also retain plasticity, and different programs (like Th1 or Th17) can be co-opted at barrier sites to suit the demands in response to re-challenge [119]. To form pathogen-specific memory populations, circulating T cells must undergo a primary T-cell response that classically involves activation within secondary lymphoid organs. In contrast, TRM cells act as first responders and are quickly reactivated upon challenge with cognate antigens. This allows them, in certain instances, to egress their niche, enter the circulation, and even repopulate distant lymphoid structures [56, 120]. Prior studies have suggested that inflammatory signals generated by pathogenic invasion of the host can “license” memory responses to the microbiota [121]. Importantly, unlike conventional TRM cells, resident TCRγδ+ T cells and CD8αα+TCRαβ+ cells bearing oligoclonal TCRs can recognize microbial products or host molecules released during stress and inflammatory responses [118]. Barrier microbiotas can hence modulate TRM responses to pathogens in different ways: they can compete for microenvironmental nutrients and accordingly limit pathogenic expansion, but they can also contribute to an environment that can control exaggerated anti-pathogen responses. Additionally, barrier bystander microbiota-reactive immune cells (including non-memory T cells) can modulate TRM responses. However, the extent and consequences of the relationship among the local microbiota, different pathogens, and the corresponding T-cell responses to such pathogens are still largely unknown.

A feature that many TRMs share is a core transcriptional program that relies heavily on Hobit (in mice), Blimp1, and Runx3 [113, 122, 123]. At a transcriptional level, both skin and intestinal TRM cells have high expression of *RORA* and *AHR*, but they display a predominant Th17 functionality program in the small intestine, while skin TRMs show more diversity, with a Th2 and Th17 bias. In allogeneic hematopoietic stem cell transplantation patients, *bona-fide* skin TRMs display a unique transcriptional signature that includes *LGALS3* as a long-term residency marker [124]. Intestinal and skin TRMs show the highest degree of clonal expansion of any organ [113], arguing that T-cell pools from these barriers have accumulated and retained much of the organism’s reactivity potential to any microbial insult over time. Microbiota-reactive CD4+ T cells from healthy individuals range from 400 to 4000/million, are enriched in gut tissues, show a memory phenotype, and express several gut-homing chemokine receptors, indicating that the T-cell repertoire of healthy individuals is reactive to intestinal commensals [56]. T cells in skin and intestinal tissues may have a differential dependence on cytokines, with IL-15 being critical for skin CD8+ TRMs maintenance and expansion, while IEL and CD4+TRMs rely more on IL-7 [125, 126]. Human intestinal CD4+ TRM express CD69 and CD161 and have a potent cytokine production potential, with a majority displaying a polyfunctional Th1-like phenotype [127]. Like in the intestinal milieu, TRM cells can also proliferate locally upon antigen encounter without exiting the skin [74, 124, 128]. Moreover, human skin CD4+ TRMs proliferate and secrete IL-17A, IL-22, IFNγ, and TNFβ when stimulated with heat-killed *S. aureus* or *C. albicans*, but not other skin commensals [128]. Interestingly, a subset of skin TRMs can re-circulate in the blood, and patients with graft versus host disease demonstrate circulating Th2/Th17-biased TRMs [129].

Responsiveness to RA, TGFβ, and SCFAs seems especially relevant for TRMs at epithelial interfaces [113, 121, 130]. RA generated by DCs from retinol is critical for inducing α4β7 expression of T cells and their recruitment to the gut [131]. Intestinal commensal *Clostridia* can modulate RA levels by suppressing the expression of the retinol dehydrogenase enzyme in epithelial cells [61], and RA signals can regulate CD8+CD103+ TRM differentiation and commitment to intestinal location during T-cell priming in mLNs [132]. Surprisingly, SFB-colonized mice contain intestinal commensals capable of directly generating RA [60]. Interestingly, RA decreases expression of CCR4 and other skin-homing molecules on mouse T cells [131], suggesting a parallel restriction of homing to the skin if RA is provided during T-cell activation under certain circumstances. TGFβ is required to retain CD8+ TRMs in the intestines, concomitantly with induction of αEβ7/α1 and CD69 [133], and TRM heterogeneity is driven by TGFβ sourced in the skin [134]. Moreover, T-cell autocrine TGFβ is one of the primary local sources in the epidermal niche, promoting antigen-specific TRM-cell accumulation and persistence [2], which can represent a positive feedback loop to amplify microbial-tolerance responses. Additionally, intestinal commensal-derived SCFAs can induce TGFβ in human intestinal epithelial cells [135], and eosinophils recruited during allergen or bacterial challenges in the intestine can also provide TGFβ to local Tregs [136]. The differences in local cell populations in skin and intestinal tissues, especially cells that can serve as APCs, will also be determinant factors in establishing a T-cell resident program.

## Conclusion

The commensal microbiome is a fundamental part of many physiological responses, strongly influencing immune cells inhabiting barrier sites that harbor rich and diverse microbial communities. Our microbiota is necessary to maintain barrier tissue homeostasis and establish proper protective and tolerant responses on immune cells, but conditions impairing barrier integrity and/or immune function can also enable the emergence of opportunistic commensal pathobionts. T cells in barrier locations constitute a unique arm of the immune system that can react in innate and adaptive fashions to signals provided by cohabiting microbiota. Barrier T cells can also integrate environmental cues in response to local commensal communities, inducing temporary T-cell states that contribute to protective, inflammatory, tolerant, or regenerative responses (Fig. 2C). Furthermore, dysregulation of T-cell responses at barrier sites is a major driver of skin and intestinal inflammatory and autoimmune disease. A better understanding of the interactivity and influence of our commensals with T cells in the skin and intestinal barriers can help devise new therapeutic approaches to restore normal T-cell function and immune health in these tissues.

## Data Availability

Not applicable.

## Abbreviations

AhR: aryl hydrocarbon receptor
AMP: anti-microbial proteins
APC: antigen presenting cell
DC: dendritic cell
ECM: extracellular matrix
HF: hair follicle
IBD: inflammatory bowel disease
IEL: intraepithelial lymphocytes
IL: interleukin
ILC: innate lymphoid cell
ILF: isolated lymphoid follicle
LN: lymph node
LP: Propria
LTA: lipoteichoic acid
MAIT: mucosal-associated T cells
MAMP: microbial-associated molecular patterns
MHC: major histocompatibility complex
NK: natural killer cell
NLR: NOD-like receptor
PSA: polysaccharide A
RA: retinoic acid
SAA: serum amyloid A proteins
SCFA: short-chain fatty acids
SFB: segmented filamentous bacteria
TCR: T cell receptor
TGFβ: transforming growing factor beta
Th: T helper cell
TLR: toll-Like receptor
TLS: tertiary lymphoid organ
Treg: regulatory T cells
TRM: tissue-resident memory T cells

## Permission to Reproduce

Figures were generated by one of the authors.
